# Rectal spacer use and overall long-term healthcare costs: payer perspective

**DOI:** 10.3389/fonc.2025.1654925

**Published:** 2025-08-12

**Authors:** James B. Yu, Ryoko Sato, Michael R. Folkert, Samir Bhattacharyya, Emmanuel Ezekekwu, Daniel A. Hamstra

**Affiliations:** ^1^ Department of Radiation Oncology, Dartmouth Hitchcock Medical Center, Hanover, NH, United States; ^2^ Boston Scientific, Marlborough, MA, United States; ^3^ Department of Radiation Oncology, University of Washington School of Medicine, Seattle, WA, United States; ^4^ Balor College of Medicine, Radiation Oncology, Houston, TX, United States

**Keywords:** prostate cancer, radiotherapy, rectal spacers, healthcare cost, healthcare utilization

## Abstract

**Introduction:**

Rectal spacers (RS), when used in prostate cancer (PCa) patients treated with radiotherapy (RT), reduce radiation dose to the rectum. While RS incur additional upfront cost, they may result in long-term cost-savings by reducing toxicity-related adverse events and associated medical costs. This study examined long-term pattern of insurer-paid healthcare costs among patients with and without polyethylene glycol hydrogel RS use.

**Methods:**

Men with PCa who received RT during 2015–2020 were identified from Medicare 5% and Merative™ MarketScan Commercial data. Multivariable generalized linear models assessed the association between RS utilization and total costs from 1-year prior to RT to 4- years after RT, controlling for age, comorbidity, RT modality, secondary cancer, baseline dysfunction, data source, year of RT, and state. Analyses were stratified by payer type (Medicare, commercial) and cost type (overall, those for specific conditions).

**Results:**

The analysis included 5,829 individuals, 270 (4.6%) of whom received RS. After controlling for covariates, costs 1-year pre-RT were significantly higher for RS patients by +$1,811 ($17,378 vs. $15,567, p=0.023), as were costs for RT (including RS) at the time of treatment by +$3,949 ($31,712 vs. $27,763, p<0.001). However, total insurer paid costs over the following 4 years post-RT were significantly lower for RS patients by $8,095 ($52,345 vs. $60,440, p=0.011). Similar patterns were observed when examining costs related to bowel, sexual, or urinary dysfunction separately.

**Conclusions:**

Patients with RS use undergoing PCa RT had significantly lower long-term overall healthcare costs despite incurring higher initial costs prior to and during RT, suggesting that upfront investment in RS may be offset by long-term savings for insurers.

## 1 Introduction

Radiotherapy (RT) is a common and effective treatment for localized prostate cancer (PCa) (1, 2) but it is known to potentially cause long-term rectal, urinary, and sexual toxicity (3, 4); including diarrhea, rectal bleeding, proctitis, urinary obstruction, incontinence, urethral strictures, and erectile dysfunction (5–8). Phase III trials have indicated similar toxicity profiles across common types of external beam radiation therapy including intensity-modulated RT (IMRT), stereotactic body RT (SBRT), and proton beam RT (9, 10). Attempts to mitigate rectal toxicity include image guidance, more precise RT techniques (11), and/or using temporary rectal spacers (RS) to increase the distance between the rectum and the prostate during RT (12–15). The use of RS among PCa patients has surged in recent years, rising from 4% in 2017 to 28% in 2021, and reaching 47% among SBRT patients in 2021 (16).

RS may reduce overall long-term healthcare costs by reducing toxicity-related complications (13, 17–19). The use of RS may also provide benefits beyond toxicity-related cost reduction, potentially influencing healthcare utilization and minimizing declines in quality of life (QOL) (13, 20, 21). The use of RS does incur additional cost at the time of RT delivery. Some studies have also reported that the use of RS may result in rare but severe toxicities, potentially leading to higher costs (22, 23). Economic evaluations of the use of RS to date have involved simulated models and focused solely on toxicity-related cost projections (24–26). Given the potential for RS to reduce healthcare costs through reduced toxicity and via other benefits, a more thorough economic analysis of healthcare utilization and costs using real-world data is needed. Therefore, this study evaluated patterns of overall long-term healthcare costs among patients with and without polyethylene glycol (PEG) hydrogel RS use from a payer perspective.

## 2 Materials and methods

### 2.1 Study type and patient population

This was a retrospective cohort study that utilized the Medicare 5% Standard Analytic Files (SAF) and the Merative™ MarketScan^®^ Commercial Database. Included were men with PCa who received RT (IMRT, SBRT, proton beam RT, brachytherapy, or any combination of these) between 2015 and 2020. Patients were required to be aged 65+ years if they were from the Medicare data and 18–64 years if they were from the MarketScan data. Only those with continuous enrollment from one year prior to RT to four years after RT were included; those undergoing prostatectomy were excluded. Medicare 100% SAF data were used for robustness analyses. While the 5% Medicare dataset used in the main analysis randomly samples five percent of all Medicare patients for all care settings, the 100% Medicare dataset includes all Medicare patients. However, the 100% Medicare sample excludes claims for office visits or from ambulatory surgery centers. Because of the retrospective nature and the use of de-identified claims data, consent and IRB approval were not required.

### 2.2 Outcomes and covariates

The primary outcome was total insurer-paid healthcare costs 1 year prior to RT, during RT (including costs associated with RS use), and up to 4 years after RT, comparing patients with and without RS. The measurement of costs was consistent across different datasets and was inflation-adjusted for 2023. Costs specifically related to bowel, sexual, or urinary dysfunction were also examined using diagnosis and procedure codes to identify them (9). A secondary outcome was the average number of medical visits (clinic visits) per year overall and specifically for bowel/sexual/urinary dysfunction.

Measures used as covariates in adjusted models included patient age, Charlson Comorbidity Index (CCI) (27) based on claims prior to RT, RT modality (IMRT, SBRT, proton beam RT, brachytherapy, or combination), the year of RT, the presence of secondary cancers (colon, rectal, bladder), bowel/sexual/urinary dysfunction at baseline (one year prior to RT), data source (Medicare 5% vs. MarketScan), and state fixed effect.

The clinical benefit of RS placement may vary by RT modality due to differences in dose distribution, treatment geometry, and proximity of adjacent organs. For example, SBRT delivers higher dose per fraction and may benefit more from RS in reducing rectal toxicity, whereas brachytherapy’s confined dose delivery might reduce the relative incremental value of RS. To account for these differences, RT modality was included as a covariate in all models.

Two sets of robustness analyses were conducted. The first robustness analysis excluded patients who underwent brachytherapy, as they were less likely to receive RS and may have higher catheter use, which could influence overall costs. The second robustness check was conducted with an additional covariate to indicate the use of androgen deprivation therapy (ADT) after PCa diagnosis as a surrogate for cancer risk, since higher-risk patients may incur greater costs and may be less likely to use a rectal spacer, as they were not included in the phase III trials. This information was available only in MarketScan data.

### 2.3 Statistical analyses

A Generalized Linear Model (GLM) with gamma distribution and log-link assessed cost differences, adjusting for covariates mentioned above. GLM regressions were also performed using propensity score–matched (PSM) samples, with patients matched on the same set of covariates described above. Results using PSM is supplemental to the main results as the number of observations included in the analysis drops substantially. *Post-hoc* robustness analyses were performed using a sample from the Medicare 100% SAF to examine whether the lack of significance found in certain differences in the original analysis was due to small sample sizes. Poisson regression was used to evaluate the number of medical visits, adjusting for the same covariates. All analyses were performed using the Instant Health Data (IHD) software (Panalgo, Boston, MA, USA) and Stata 18.0 (StataCorp LLC, Texas, USA).

## 3 Results

### 3.1 Patient population

A total of 5,829 PCa patients were identified as undergoing RT, including 270 (4.6%) who received RS. Those receiving RS were slightly older (69.3vs. 68.2, p=0.038) with a lower CCI (2.4 vs. 2.6, p=0.03) than those treated with RT without RS. The race distribution among the Medicare population (the only patients for whom race data were available) was not statistically significantly different between groups. Patients who received RS were more likely to undergo SBRT (20.7% vs. 8.5%, p<0.001) and less likely to receive IMRT (37.8% vs. 57.5%, p<0.001, **Table 1**) than those treated without RS. Patients who underwent brachytherapy had the lowest likelihood of receiving RS at 1.4% (table not shown).

**Table 1:** Summary statistics.

	No rectal spacer (n=5,559)		Rectal spacer (n=270)		P-value
*Continuous variables*	Mean	SD	Mean	SD	
Age at RT	68.2	8.3	69.3	7.6	0.038
CCI at RT	2.6	1.4	2.4	1.1	0.033
*Categorical variables*	#	%	#	%	
Data source					
Medicare	4,355	78.3	224	83.0	0.071
MarketScan	1,204	21.7	46	17.0	
RT modality					
IMRT	3,195	57.5	102	37.8	<0.001
SBRT	474	8.5	56	20.7	
Proton	295	5.3	75	27.8	
Brachy	1,093	19.7	16	5.9	
Combo	502	9.0	21	7.8	
Year of RT					
2015	1,515	27.3	0	0.0	<0.001
2016	1,380	24.8	12	4.4	
2017	1,328	23.9	71	26.3	
2018	1,336	24.0	187	69.3	
Region					
Midwest	1,262	22.9	34	12.8	0.001
Northeast	1,074	19.5	52	19.6	
South	2,302	41.8	131	49.3	
West	871	15.8	49	18.4	
Secondary cancer	527	9.5	21	7.8	0.35
Base bowel diagnosis (1 year)	278	5.0	15	5.6	0.68
Base sexual diagnosis (1 year)	746	13.4	32	11.9	0.46
Base urinary diagnosis (1 year)	2,318	41.7	94	34.8	0.025
Race (only in Medicare 5%)					
White	3,540	81.3	186	83.0	0.082
Black	550	12.6	19	8.5	
Other	265	6.1	19	8.5	

CCI, Charlson Comorbidity Index; IMRT, intensity modulated radiation therapy; RT, radiotherapy; SBRT, stereotactic body radiation therapy.

Sample: Medicare 5% + MarketScan, continuous enrollment 1 year prior to RT, continuous enrollment 4 years post RT, no prostatectomy.

### 3.2 Costs

**Figure 1:**
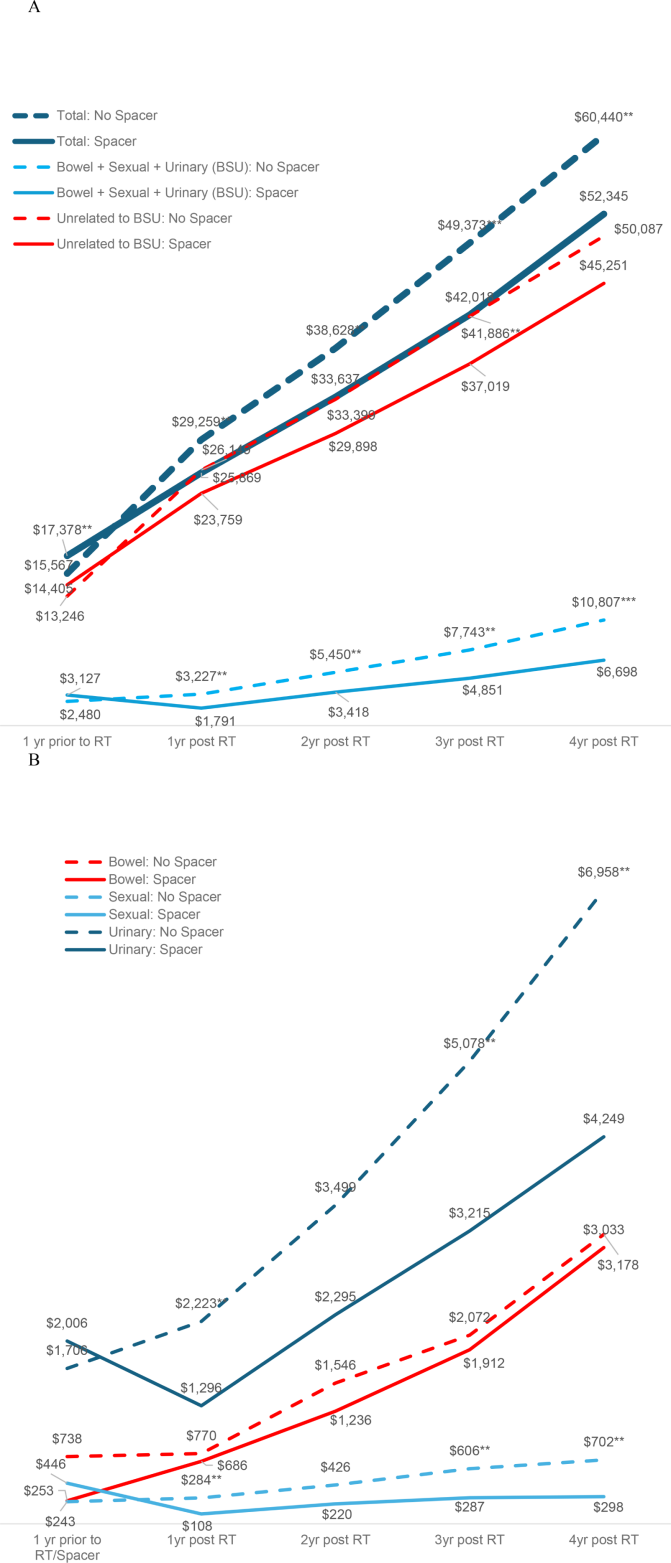
Costs of care over time by use of a rectal spacer during radiotherapy. Panel **(A)** Total costs, costs related to bowel/sexual/urinary dysfunction, and other unrelated costs. Panel **(B)** Costs of Care for Bowel, Sexual, or Urinary Dysfunction.

After controlling for covariates in the regression, estimated costs during the 1-year period pre-RT were significantly higher for RS patients by +$1,811 ($17,378 vs. $15,567, p=0.023), as were costs for RT, including costs associated with RS use, by +$3,949 ($31,712 vs. $27,763, p<0.001). However, over four years post-RT total insurer-paid costs were significantly lower for RS patients by -$8,095 ($52,345 vs. $60,440, p=0.011, **Table 2**, **Figure 1** Panel A). The pattern of lower costs for those treated with RS was consistent after applying PSM. However, the results were not statistically significant in the Medicare 5% and MarketScan datasets, likely due to the small sample size (**Table 3**). Alternatively, the findings were both consistent and statistically significant when using the Medicare 100% dataset. Given that RS use was lowest among patients who underwent brachytherapy, **Table 4** presents total cost of care results excluding these patients, demonstrating the consistency of the findings. Furthermore, the result was robust and consistent even after the use of ADT was controlled for in the regression analyses in MarketScan data (**Supplementary Table 1**). Costs specific to bowel/sexual/urinary-related conditions were -$4,109 lower at 4-years post-RT in those with RS as compared to those without ($10,807 versus $6,698, p=0.009, **Table 2**).

**Table 2:** Total cost of care by spacer status.

	No rectal spacer (n=5,559)	Rectal spacer (n=270)	Difference	P-value
All costs of care				
1 year prior to RT	$15,567	$17,378	+$1,811	0.023
	($15097 to $16037)	($15768 to $18987)		
RT + RS	$27,763	$31,712	+$3,949	<0.001
	($27214 to $28311)	($29646 to $33779)		
1 year post RT	$29,259	$25,869	-$3,390	0.034
	($28559 to $29959)	($22973 to $28765)		
2 years post RT	$38,628	$33,637	-$4,991	0.017
	($37649 to $39607)	($29898 to $37375)		
3 years post RT	$49,373	$42,018	-$7,356	0.004
	($47971 to $50776)	($37405 to $46630)		
4 years post RT	$60,440	$52,345	-$8,095	0.011
	($58694 to $62186)	($46605 to $58086)		
Bowel, sexual, urinary-related costs				
1 year prior to RT	$2,480	$3,127	+$647	0.25
	($2180 to $2779)	($1893 to $4362)		
1 year post RT	$3,227	$1,791	-$1,437	0.036
	($2842 to $3613)	($834 to $2748)		
2 years post RT	$5,450	$3,418	-$2,031	0.03
	($4825 to $6075)	($1991 to $4846)		
3 years post RT	$7,743	$4,851	-$2,892	0.02
	($6962 to $8523)	($2965 to $6736)		
4 years post RT	$10,807	$6,698	-$4,109	0.009
	($9372 to $12242)	($4245 to $9152)		
All other costs				
1 year prior to RT	$13,246	$14,405	+$1,159	0.096
	($12844 to $13649)	($13023 to $15788)		
1 year post RT	$26,149	$23,759	-$2,390	0.102
	($25498 to $26801)	($21101 to $26417)		
2 years post RT	$33,399	$29,898	-$3,501	0.059
	($32544 to $34253)	($26561 to $33236)		
3 years post RT	$41,886	$37,019	-$4,867	0.026
	($40664 to $43108)	($33047 to $40990)		
4 years post RT	$50,087	$45,251	-$4,836	0.065
	($48551 to $51624)	($40423 to $50080)		

RT, radiotherapy. RS, rectal spacer.

Values are mean and (95% confidence interval) from Generalized Linear Models (GLMs) controlling for covariates (age, Charlson Comorbidity Index, modality, secondary cancer, baseline bowel/sexual/urinary dysfunction, data source, year of radiation therapy, and state fixed effects). The values for “Bowel, Sexual, Urinary-related costs” and “All other costs” do not sum to “All Costs of Care” because each is produced by a separate GLM. Costs for the 1-year period prior to RT exclude the cost of the spacer. All costs “post” RT include all costs after the initiation of RT, excluding the cost of RT itself, and are cumulative.

**Table 3:** Association between spacer use and all costs of care, using propensity score matching.

All Costs of Care				
	No rectal spacer	Rectal spacer	Difference	P-value
Panel A: Medicare 5% + MarketScan (N=469)				
1 year prior to RT	$14,116 ($12369 to $15862)	$15,833 ($14455 to $17211)	+$1,717	0.114
RT + RS	$30,083 ($27811 to $32355)	$35,921 ($33293 to $38548)	+$5,838	<0.001
1 year post RT	$29,794 ($26717 to $32871)	$30,091 ($26856 to $33327)	+$297	0.897
2 years post RT	$37,368 ($33466 to $41271)	$37,622 ($33347 to $41898)	+$254	0.934
3 years post RT	$45,933 ($40644 to $51222)	$44,137 ($39251 to $49022)	-$1,796	0.629
4 years post RT	$56,598 ($49670 to $63526)	$53,785 ($47753 to $59818)	-$2,813	0.550
Panel B: Medicare 100% (N=11,117)				
1 year prior to RT	$7,882 ($7221 to $8543)	$11,001 ($10570 to $11431)	+$3,119	<0.001
RT + RS	$18,296 ($17743 to $18849)	$21,471 ($21266 to $21675)	+$3,175	<0.001
1 year post RT	$18,855 ($17514 to $20197)	$16,652 ($15958 to $17345)	-$2,203	0.001
2 years post RT	$26,160 ($24221 to $28099)	$22,176 ($21345 to $23007)	-$3,984	<0.001
3 years post RT	$33,366 ($30928 to $35804)	$28,337 ($27302 to $29372)	-$5,029	<0.001
4 years post RT	$41,114 ($38201 to $44067)	$34,783 ($33574 to $35993)	-$6,331	<0.001

RT, radiotherapy.

Propensity score matching was based on covariates: age, Charlson Comorbidity Index, modality, secondary cancer, baseline bowel/sexual/urinary dysfunction, data source, year of radiation therapy, and region. The 100% Medicare dataset includes all Medicare patients, but excludes claims for office visits or from ambulatory surgery centers. Values are mean and (95% confidence interval) from Generalized Linear Models (GLMs) controlling for covariates (age, Charlson Comorbidity Index, modality, secondary cancer, baseline bowel/sexual/urinary dysfunction, data source, year of radiation therapy, and state fixed effects). Costs for the 1-year period prior to RT exclude the cost of the spacer. All costs “post” RT include all costs after the initiation of RT, excluding the cost of RT itself, and are cumulative.

**Table 4:** Total cost of care for RT patients excluding brachytherapy.

	No rectal spacer (n=4,466)	Rectal spacer (n=254)	Difference	P-value
All costs of care				
1 year prior to RT	$16,106	$17,378	+$1,272	0.044
	($15567 to $16645)	($15768 to $18987)		
RT + RS	$31,819	$35,580	+$3,761	<0.001
	($31140 to $32497)	($33333 to $37826)		
1 year post RT	$32,876	$29,496	-$3,380	0.065
	($32072 to $33679)	($26143 to $32849)		
2 years post RT	$42,550	$37,545	-$5,005	0.032
	($41446 to $43653)	($33331 to $41760)		
3 years post RT	$53,795	$46,521	-$7,274	0.012
	($52209 to $55381)	($41301 to $51740)		
4 years post RT	$65,609	$56,964	-$8,645	0.016
	($63613 to $67605)	($50501 to $63428)		

RT, radiotherapy. RS, rectal spacer.

Values are mean and (95% confidence interval) from Generalized Linear Models (GLMs) controlling for covariates (age, Charlson Comorbidity Index, modality, secondary cancer, baseline bowel/sexual/urinary dysfunction, data source, year of radiation therapy, and state fixed effects). Costs for the 1-year period prior to RT exclude the cost of the spacer. All costs “post” RT include all costs after the initiation of RT, excluding the cost of RT itself, and are cumulative.

The pattern in bowel/sexual/urinary-related costs was primarily driven by urinary-related costs that were insignificantly higher among patients with RS prior to RT ($2,006 vs. $1,706, p=0.404) but significantly lower at 3- and 4-years post RT ($3,215 vs. $5,078, p=0.033 at 3-years; $4,249 vs. $6,958, p=0.017 at 4-years, **Table 5**, **Figure 1** Panel B). Sexual-related costs followed a similar pattern but with a smaller cost difference; there were higher costs among RS patients prior to RT ($446 vs. $243, p=0.093) but lower costs post RT ($287 vs. $606, p=0.046 at 3-years; $298 vs. $702, p=0.016 at 4-years). Bowel-related costs were also lower among RS patients post RT, but not significantly different from non-RS patients ($3,033 vs. $3,178, p=0.884 at 4-years). However, when costs were examined using the larger Medicare 100% sample, which included 114,777 PCa patients treated without RS and 8,740 treated with RS, total costs were significantly lower at 4-years in those treated with RS by -$3,092 ($38,523 vs. $35,431, p<0.001 at 4-years), and bowel-related costs were also significantly lower at 2- and 4-years post RT among patients who received RS ($617 vs. $823, p=0.022 at 2-years; $1,345 vs. $1,649, p=0.030 at 4-years, **Supplementary Table 2**).

**Table 5:** Bowel, sexual, and urinary-related costs by rectal spacer status.

	No rectal spacer (n=5,559)	Rectal spacer (n=270)	Difference	P-value
Bowel-related costs only				
1 year prior to RT	$738	$253	-$485	0.054
	($254 to $1223)	(-$22 to $528)		
1 year post RT	$770	$686	-$84	0.84
	($511 to $1030)	(-$77 to $1449)		
2 years post RT	$1,546	$1,236	-$310	0.55
	($1197 to $1895)	($362 to $2111)		
3 years post RT	$2,072	$1,912	-$160	0.83
	($1691 to $2452)	($574 to $3250)		
4 years post RT	$3,178	$3,033	-$145	0.88
	($2373 to $3982)	($1080 to $4985)		
Sexual-related costs only				
1 year prior to RT	$243	$446	+$203	0.093
	($164 to $323)	($131 to $761)		
1 year post RT	$284	$108	-$175	0.045
	($177 to $390)	($0.80 to $216)		
2 years post RT	$426	$220	-$206	0.090
	($285 to $567)	($40 to $400)		
3 years post RT	$606	$287	-$319	0.046
	($433 to $778)	($62 to $511)		
4 years post RT	$702	$298	-$404	0.016
	($531 to $872)	($79 to $517)		
Urinary-related costs only				
1 year prior to RT	$1,706	$2,006	+$300	0.404
	($503 to $1090)	($1247 to $2764)		
1 year post RT	$2,223	$1,296	-$926	0.042
	($1974 to $2471)	($639 to $1953)		
2 years post RT	$3,400	$2,295	-$1,204	0.070
	($3139 to $3858)	($1264 to $3327)		
3 years post RT	$5,078	$3,215	-$1,862	0.033
	($4587 to $5596)	($1888 to $4543)		
4 years post RT	$6,958	$4,249	-$2,710	0.017
	($6230 to $7686)	($2557 to $5940)		

RT, radiotherapy.

Values are mean and (95% confidence interval) from Generalized Linear Models (GLMs) controlling for covariates (age, Charlson Comorbidity Index, modality, secondary cancer, baseline bowel/sexual/urinary dysfunction, data source, year of radiation therapy, and state fixed effects). Costs for the 1-year period prior to RT exclude the cost of the spacer. All costs “post” RT include all costs after the initiation of RT, excluding the cost of RT itself, and are cumulative.

When analyzed separately for each RT modality, patients who received RS generally had higher costs one year prior to RT and during RT (including the cost of the RS). However, four-year post-RT costs were lower, although the difference was not statistically significant when broken down by individual RT modality (**Supplementary Figure 1**, Panel A). Given the modest sample sizes, the analysis was repeated using Medicare 100% data and patients with RS use had significantly lower costs four years post-RT among those who underwent SBRT, IMRT, or a combination of multiple RT modalities (**Supplementary Figure 1**, Panel B).

The overall cost difference was most pronounced among commercially-insured (MarketScan) patients. The cost difference at 4 years among MarketScan patients was -$34,995 ($48,210 for patients with RS vs. $83,205 for those without, p<0.001, **Supplementary Table 3**), while a similar but not significant difference was observed among Medicare patients (-$4,284 lower for patients with RS, p=0.17). Within MarketScan patients, the cost savings associated with the use of RS occurred at each of the 4 years post-RT (1-year post-RT = -$22,477, p=0.001; 2-years post-RT = -$27,055, p<0.001; 3-years post-RT = -$31,813, p<0.001; 4-years post-RT = -$34,995, p<0.001; **Supplementary Table 3**). It should be noted that in general, costs for each time period and group were typically 1.5 to 3 times higher in MarketScan patients than in Medicare patients.

### 3.3 Procedures

The common procedures related to bowel/sexual/urinary dysfunction during the 4-year period post-RT are enumerated in **Table 6** and **Supplementary Table 4**, with the most common procedures with the greatest differences noted for “Insertion of temporary indwelling bladder catheter” (7.7 without RS vs. 0.9 with RS per 1000 person-years), “Colonoscopy, flexible; with control of bleeding, any method” (6.3 without RS vs. 1.9 with RS per 1000 person-years), and “Injection of corpora cavernosa with pharmacologic agent(s)” (5.0 without RS vs. 7.4 with RS per 1000 person-years). Overall, there were fewer bowel- and urinary-related procedures observed among RS patients during the 4-year post-RT period: 10.2 with RS vs 14.9 without RS per 1000 person-years for bowel-related procedures (difference -4.7 per 1000 person-years); 17.6 with RS vs 27.5 without RS per 1000 person-years for urinary-related procedures (difference -9.9 per 1000 person-years), while the number of sexual-related procedures was low for both RS and non-RS patients: 7.4 with RS vs 6.8 without RS per 1000 person/years (difference +0.6 per 1000 person-years) (**Supplementary Table 4b**). Two CPT codes presented in **Table 6**—46600 and 96402—were not included in the primary cost analysis, which strictly adhered to the categorization defined by Pan et al. (2018), and were therefore initially classified as “codes unrelated to bowel, sexual, or urinary dysfunction.” However, code 46600 was subsequently reclassified as a “bowel, sexual, urinary-related code,” specifically within the bowel-related domain, based on clinical judgment. In contrast, code 96402 was retained in the “unrelated” category despite its potential relevance to the sexual domain, as it represents part of the initial treatment rather than an intervention addressing post-radiotherapy dysfunction.

**Table 6:** Common procedures (CPT) in 4 years post-radiation therapy by rectal spacer status.

		# per year per 1000		
CPT code	Description	No RS	RS	Total
Bowel, sexual, urinary-related codes				
51702	Insertion of temporary indwelling bladder catheter; simple (e.g., Foley).	7.7	0.9	7.4
45382	Colonoscopy, flexible; with control of bleeding, any method.	6.3	1.9	6.0
54235	Injection of corpora cavernosa with pharmacologic agent(s) (e.g., papaverine, phentolamine).	5.0	7.4	5.1
45334	Sigmoidoscopy, flexible; with control of bleeding, any method.	4.2	3.7	4.2
52224	Cystourethroscopy, with fulguration (including cryosurgery or laser surgery) and/or resection of a small bladder tumor.	3.5	3.7	3.5
52281	Cystourethroscopy, with calibration and/or dilation of urethral stricture or stenosis, with or without meatotomy.	3.5	1.9	3.4
46600*	Anoscopy; diagnostic, with or without collection of specimen(s) by brushing or washing (separate procedure).	2.3	0.9	2.2
99183	Physician attendance and supervision of hyperbaric oxygen therapy, per session.	2.2	1.9	2.2
52276	Cystourethroscopy with direct vision internal urethrotomy.	1.3	1.9	1.3
52601	Transurethral resection of prostate; complete (including vasectomy, meatotomy, cystourethroscopy, urethral calibration).	1.3	0.0	1.2
52001	Cystourethroscopy with irrigation and evacuation of multiple obstructing clots.	1.1	1.9	1.1
51701	Insertion of non-indwelling bladder catheter (e.g., straight catheterization for residual urine).	1.0	0.9	1.0
53445	Insertion of artificial urinary sphincter, including placement of pump, reservoir, and cuff.	0.9	0.0	0.9
52214	Cystourethroscopy with fulguration of a lesion (excluding tumors or malignancies).	0.7	1.9	0.8
54405	Insertion of multi-component, inflatable penile prosthesis, including placement of pump, cylinders, and reservoir.	0.8	0.0	0.7
52630	Transurethral resection; residual or regrowth of obstructive prostate tissue.	0.7	0.0	0.6
64581	Incision for implantation of neurostimulator electrode array; sacral nerve (transforaminal placement).	0.5	0.0	0.5
53600	Dilation of urethral stricture by passage of sound or urethral dilator, male; initial.	0.5	0.0	0.5
52275	Cystourethroscopy with internal urethrotomy; female.	0.4	0.0	0.4
51703	Insertion of temporary indwelling bladder catheter; complicated (e.g., altered anatomy, fractured catheter/balloon).	0.4	0.0	0.3
51040	Vesicostomy (e.g., cutaneous vesicostomy, vesicostomy with skin flap).	0.3	0.9	0.3
Codes unrelated to bowel, sexual, urinary dysfunction				
20610	Arthrocentesis, aspiration, and/or injection of a major joint or bursa (e.g., shoulder, hip, knee joint), without ultrasound guidance.	44.4	60.2	45.1
17003	Destruction of premalignant lesions (e.g., actinic keratoses); second through 14 lesions, each.	27.5	27.8	27.5
90686	Influenza virus vaccine, quadrivalent (IIV4), split virus, preservative-free, 0.5 mL dosage, for intramuscular use.	13.5	10.2	13.4
99214	Office or other outpatient visit for the evaluation and management of an established patient, moderate to high severity.	10.8	4.6	10.6
86140	C-reactive protein; quantitative.	9.8	4.6	9.6
90662	Influenza virus vaccine, quadrivalent (IIV4), split virus, preservative-free, enhanced immunogenicity via increased antigen content, for intramuscular use.	8.1	9.3	8.1
90682	Influenza virus vaccine, quadrivalent (RIV4), derived from recombinant DNA, hemagglutinin (HA) protein only, preservative and antibiotic free, for intramuscular use.	7.9	9.3	8.0
92004	Ophthalmological services: medical examination and evaluation with initiation of diagnostic and treatment program; comprehensive, new patient, one or more visits.	5.9	6.5	6.0
95811	Sleep study: polysomnography; 6 or more parameters including EEG, sleep staging, and ventilation, attended by a technologist.	5.4	6.5	5.4
71046	Radiologic examination, chest; 2 views.	4.9	6.5	5.0
73502	Radiologic examination, hip, unilateral, with pelvis when performed; 2–3 views.	4.6	5.6	4.7
96402*	Chemotherapy administration, subcutaneous or intramuscular; hormonal anti-neoplastic.	4.4	2.8	4.3
93312	Echocardiography, transesophageal, real-time with image documentation (2D), including probe placement, image acquisition, interpretation, and report.	4.3	1.9	4.2
97116	Therapeutic procedure, one or more areas, each 15 minutes; gait training (includes stair climbing).	4.3	1.9	4.2
70551	Magnetic resonance imaging (MRI) of the brain (including brain stem); without contrast material.	3.3	4.6	3.4
87591	Infectious agent detection by nucleic acid (DNA or RNA); Neisseria gonorrhoeae, amplified probe technique.	3.4	3.7	3.4
92025	Computerized corneal topography, unilateral or bilateral, with interpretation and report.	2.9	7.4	3.1
88120	Cytopathology, *in situ* hybridization (e.g., FISH), urinary tract specimen with morphometric analysis; 3–5 probes.	2.5	2.8	2.5
88360	Morphometric analysis, tumor immunohistochemistry (e.g., HER-2/neu), quantitative or semiquantitative, per specimen; manual.	2.4	1.9	2.4

RS, rectal spacer.

*not included in cost analysis as described by Pan et al. (2018).

### 3.4 Clinic visits

The annual average number of clinic visits was similar during the 1-year period prior to RT (32.97 vs 32.98, p=0.99), but over the 4 years of follow-up, patients with RS averaged 0.87 fewer visits per year than patients without RS (29.6 versus 28.8, p=0.018, **Supplementary Table 5**). This difference included significantly fewer urinary-related visits (1.42 versus 1.27, p=0.036) and visits unrelated to bowel/sexual/urinary conditions (27.7 versus 27.0, p=0.042).

## 4 Discussion

This analysis of real-world data from 1 year prior to and 4 years after RT with or without RS provides a robust sample to analyze health-related costs from the payer perspective. To date, three different RS devices have been approved by the FDA for use during prostate RT, with available phase III trials data limited to follow-up durations of 33, 6, and 6 months, respectively. All 3 devices demonstrated substantial reductions in radiation dose delivered to critical normal structures in the pelvis with a focus on rectal sparing (13–15). However, the long-term cost-benefit of these devices has been less robustly analyzed. Of note, the primary analysis group presented in this study – consisting of 270 patients treated with RT and RS and followed for 4 years – provides twice the cumulative follow-up of all three prospective phase III trials combined for patients with RS. Furthermore, the expanded analysis using the Medicare 100% population, which includes 8,740 patients treated with RT and RS and followed for 4-years, represents a >60-fold increased cumulative follow-up.

In this observational analysis of Medicare and commercial insurance data from 2015 to 2020, patients who received rectal spacers (RS) had lower overall healthcare costs over the 4-year period following RT. This was observed despite showing that the costs of RT (including RS) as well as costs during the 1-year period prior to RT were significantly higher among patients who received RS. Lower overall healthcare costs following RT among the RS cohort were consistently observed across multiple analyses (1): using PSM to reduce selection bias in RS use (2), excluding patients who underwent brachytherapy—who were least likely to receive RS and may have higher catheter use—and (3) controlling for ADT use as a proxy for cancer severity in the MarketScan sample. Cost differences were observed in both commercial and Medicare populations; however, they were larger in commercial populations, which may be partly due to higher costs generally in commercial versus Medicare plans (28, 29). It is notable that, despite the high costs billed to commercial insurance, commercially insured RS patients incurred post-RT expenses comparable to those of Medicare RS patients (e.g., $40,268 for Medicare vs. $39,478 for MarketScan over three years post-RT; $50,692 for Medicare vs. $48,210 for MarketScan over four years post-RT). However, the number of observations for MarketScan RS patients was small (n=46), requiring cautious interpretation. As such, these findings should be considered exploratory and not definitive.

Other studies have attempted to estimate the cost-effectiveness of the use of RS, but all used models or simulation instead of real-world data and focused solely on toxicity-related costs without assessing overall costs. Studies by Levy et al. and by Venneste et al. used Markov Models with 5-year horizons. These studies estimated higher incremental costs for patients using RS over the 5-year period ($3578 and €1540, respectively) (24, 26). However, the costs considered post-RT were limited. For example, Venneste et al. considered lab tests, medication for diarrhea, transfusions, sigmoidoscopies, and specialist consultations only, depending on level of toxicity (26). Levy et al. considered post-RT costs associated with remission, intestinal/urinal toxicity, and erectile dysfunction (24). In an additional analysis, Hutchinson et al. estimated an incremental cost of RS of only $518 over 10-years following IMRT using a decision tree model, but also considered only costs related to toxicity (25). It is notable that even without considering all costs, Hutchinson et al. found the use of RS for patients receiving dose escalated SBRT to be immediately cost-effective due to savings from reduced toxicity. In contrast, the current study provides real-world evidence from national Medicare and commercial claims databases, encompassing all-cause insurer-paid costs over a 5-year window (1 year pre-RT and 4 years post-RT). This broader scope includes not only bowel, urinary, and sexual dysfunction-related costs, but also general medical expenses that may reflect downstream effects of treatment-related morbidity or patient frailty. While prior models assumed a constrained cost impact from RS based on toxicity outcomes alone, our findings demonstrate that RS use was consistently associated with lower long-term healthcare costs—particularly among commercially insured patients—even when accounting for the higher upfront cost of the device and RT delivery. This comprehensive cost accounting underscores the potential for RS to yield economic value beyond toxicity mitigation, a contribution that simulation models to date have not been able to fully capture.

The primary analysis performed here analyzed all health care costs for those treated with RT with and without RS to provide the most comprehensive and unbiased approach. Planned secondary analyses broke down by costs most likely to be related to prostate cancer and/or treatment (bowel, urinary, or sexual events) as compared to other costs less likely to be directly related to prostate cancer or treatment. Interestingly, and somewhat unexpectedly, the greatest difference in bowel, urinary, and sexual costs was for urinary costs. In the Medicare 5% samples (which is a much smaller population of only 5,839 men but which covers all billing and coding costs), total costs, costs related to urinary/bowel/sexual side effects, and unrelated costs were all lower in those with RS (although non-related costs were only lower through 3 years). When broken down further, both urinary and sexual costs were lower in those with RS while bowel costs were not. We hypothesized that one reason that bowel costs were not significantly lower could be the much smaller sample size in the Medicare 5% random sample as compared to the 100% Medicare data set. The larger Medicare 100% dataset includes 114,777 patients with RT without RS and 8,744 patients with RS, but this dataset has less complete coverage of billing codes as neither office visits nor surgical center codes are captured (**Supplementary Table 2**). In this larger Medicare data, all costs, bowel costs, and urinary costs were all lower with the use of RS, with the total cost difference after 4-years (-$3,092) comparable to the added cost of RS at the time of RT (+$3,131). The observed lower total cost over 4-years in the Medicare 100% data set (-$3,092) is also notable in that this group of patients who later were treated with RS and RT had utilized substantially greater resources in the year prior to RT (+$2,998), as such, the reduction at 4 years may actually have been significantly greater than the observed reduction by almost 50% (-$3,092 reduced cost at 4 years and +$2,998 greater cost prior to RT = difference -$6,090).

The current study adds to the literature in this space by using real-world data and considering all costs, in addition to examining costs reasonably attributed to RT and/or RS toxicity. This study observed differences not just in costs attributable to dysfunction that may be related to bowel/sexual/urinary toxicity, but in other and unrelated costs as well as overall costs. It is unclear why costs for services not directly related to bowel/urinary/sexual dysfunction were higher following RT in those who did not have RS as compared to those with had RT with RS. One possibility is that greater treatment-related side-effects such as those related to bowel/urinary/sexual dysfunction post-RT led to more frequent medical visits and a corresponding increase in other costs not typically associated with PCa or the toxicity directly attributable to treatment. Another explanation for higher costs in non-RS patients is that despite adjustment by age and comorbid conditions, that the non-RS cohort was enriched with patients who were more likely to fit a “high need” Medicare phenotype (i.e., those with heart disease, skilled nursing facility or home care, hospitalizations, and Alzheimer’s disease and related dementias with functional dependency) (30). However, the finding of lower healthcare costs for patients without RS placement in the year prior to RT somewhat refutes that explanation. One would expect a “high need” phenotype to have had higher healthcare costs in the year prior to RT in addition to the years after.

Beyond cost considerations, the use of rectal spacers has been associated with improvements in patient-reported quality of life (QoL). Phase III trial data and pooled prospective studies have demonstrated that RS placement reduces bowel toxicity, which translates into meaningful long-term QoL benefits, particularly in bowel and urinary domains. For example, Hamstra et al. reported improved sexual QoL outcomes in patients receiving intensity-modulated radiation therapy (IMRT) with a rectal spacer compared to those without (21). Similarly, Pham et al. showed favorable long-term patient-reported QoL metrics following SBRT in patients who received hydrogel spacers (20). While QoL outcomes were not directly measured in our claims-based study, these prior findings suggest that the reduction in toxicity-related complications observed here likely confers parallel benefits in patient experience and daily functioning. Future real-world studies integrating clinical and patient-reported outcomes would help validate these relationships more directly.

The strengths of this study include the use of two large national datasets that cover all ages 18 years and over and that provide real-world data with 4 years of follow-up. Also, in addition to considering costs specific to rectal, urinary, and sexual toxicity, this study examines overall costs. Limitations of this study include those inherent to the nature of claims-based analyses. Namely, claims reflect billing and are subject to human error in coding and/or completeness. And, while these data allow for the examination of association, inferences on causation cannot be made. In particular, there is the potential for unmeasured confounding and selection bias, as the decision to use a rectal spacer was not randomized and may have been influenced by clinical judgment, patient preferences, tumor characteristics, provider experience, or institutional protocols. These factors - many of which are not captured in claims data - may correlate with both the likelihood of RS utilization and downstream healthcare utilization or cost. Although measurable confounders were adjusted including age, comorbidity, baseline dysfunction, and RT modality, and conducted propensity score matching and sensitivity analyses, residual confounding cannot be excluded. Future studies with more granular clinical data or randomized designs would be needed to better isolate the causal impact of RS use on long-term outcomes. Finally, the retrospective design may introduce selection bias, as patients were not randomly assigned to receive RS.

Despite these limitations, this study found that although patients undergoing PCa RT with RS had higher baseline healthcare costs, they subsequently experienced significantly lower overall healthcare costs over the 4 years after RT, even after accounting for the added cost of the RS. Some of these differences may be due to underlying patient selection factors that could not be captured in claims data and thus not controlled in the analyses. In addition, this claims-based analysis cannot directly account for reduced toxicity and improved QOL that has been demonstrated with the use of RS. Nonetheless, these findings highlight the potential beneficial economic impact of RS placement from a payer perspective.

## Data Availability

The data analyzed in this study is subject to the following licenses/restrictions: Data are not available for sharing because of data-use agreements with CMS and Merative. However, data can be made available to reasonable requests upon the approval of both entities. Requests to access these datasets should be directed to Research Data Assistance Center (ResDAC), resdac@umn.edu; Merative MarketScan Research databases, info@merative.com.
