# Supplementary material for: Rectal spacer use and overall long-term healthcare costs: payer perspective
Source: Front Oncol. 2025 Aug 12;15:1654925. doi: 10.3389/fonc.2025.1654925 (PMC12381875; doi:10.3389/fonc.2025.1654925)
Supplement: Supplementary file 1 [file Table1.docx]

Supplementary Material

# Supplementary Tables


**Supplemental Table 1**. Association Between Spacer Use and Cost of Care, with an Additional Covariate of Medication Use in MarketScan Data

|  | Total costs of care, Coefficient (95%CI) | | | | | |
| --- | --- | --- | --- | --- | --- | --- |
|  | 1 year prior to RT | RT + RS | 1 year post RT | 2 years post RT | 3 years post RT | 4 years post RT |
| RS | 0.237** | 0.194 | -0.585*** | -0.605*** | -0.596*** | -0.550*** |
|  | (0.002 to 0.472) | (-0.082 to 0.469) | (-0.932 to -0.239) | (-0.925 to -0.286) | (-0.901 to -0.290) | (-0.855 to -0.246) |
| Use of anti-hormonal therapy drugs | 0.055 | 0.081 | 0.079 | 0.085 | 0.145 | 0.166** |
|  | (-0.086 to 0.197) | (-0.025 to 0.187) | (-0.061 to 0.218) | (-0.065 to 0.234) | (-0.011 to 0.301) | (0.010 to 0.322) |
| N | 1172 | 1172 | 1172 | 1172 | 1172 | 1172 |

RT, radiotherapy; RS, rectal spacer

Positive coefficients indicate that the spacer use (RS) is associated with higher costs, and negative coefficients indicate that the spacer use is associated with lower costs. Values are estimated coefficient from Generalized Linear Models (GLMs) controlling for covariates (age, Charlson Comorbidity Index, modality, secondary cancer, baseline bowel/sexual/urinary dysfunction, data source, year of radiation therapy, and state fixed effects). Costs for the 1-year period prior to RT exclude the cost of the spacer. All costs “post” RT include all costs after the initiation of RT, excluding the cost of RT itself, and are cumulative.

**Supplemental Table 2**. Cost of Care in Medicare 100% Data

|  | No Spacer (n=114,777) | Spacer (n=8,740) | Difference | p-value |
| --- | --- | --- | --- | --- |
| All Costs of Care | |  |  |  |
| 1 year prior to RT | $7,983 | $10,981 | +$2,998 | <0.001 |
|  | ($7095 to $8062) | ($10728 to $11234) |  |  |
| RT + Spacer | $19,147 | $22,278 | +$3,131 | <0.001 |
|  | ($19096 to $19199) | ($22080 to $22476) |  |  |
| 1 year post RT | $18,256 | $16,562 | -$1,694 | <0.001 |
|  | ($18151 to $18361) | ($16109 to $17015) |  |  |
| 2 years post RT | $24,644 | $22,368 | -$2,276 | <0.001 |
|  | ($24485 to $24803) | ($21756 to $22980) |  |  |
| 3 years post RT | $31,207 | $28,711 | -$2,496 | <0.001 |
|  | ($30995 to $31418) | ($27924 to $29498) |  |  |
| 4 years post RT | $38,523 | $35,431 | -$3,092 | <0.001 |
|  | ($38252 to $38794) | ($34454 to $36407) |  |  |
|  |  |  |  |  |
| Bowel-Related Costs | |  |  |  |
| 1 year prior to RT | $374 | $174 | -$200 | <0.001 |
|  | ($313 to $435) | ($106 to $242) |  |  |
| 1 year post RT | $384 | $359 | -$25 | 0.68 |
|  | ($354 to $413) | ($245 to $472) |  |  |
| 2 year post RT | $823 | $617 | -$206 | 0.022 |
|  | ($781 to $865) | ($467 to $767) |  |  |
| 3 year post RT | $1 to192 | $1 to016 | -$175 | 0.15 |
|  | ($1140 to $1243) | ($803 to $1229) |  |  |
| 4 year post RT | $1 to649 | $1 to345 | -$304 | 0.030 |
|  | ($1583 to $1715) | ($1104 to $1586) |  |  |
|  |  |  |  |  |
| Sexual-Related Costs | |  |  |  |
| 1 year prior to RT | $159 | $365 | +$206 | <0.001 |
|  | ($139 to $179) | ($277 to $452) |  |  |
| 1 year post RT | $184 | $153 | -$30 | 0.18 |
|  | ($171 to $197) | ($115 to $192) |  |  |
| 2 year post RT | $265 | $250 | -$14 | 0.63 |
|  | ($250 to $280) | ($196 to $304) |  |  |
| 3 year post RT | $345 | $302 | -$43 | 0.18 |
|  | ($328 to $362) | ($247 to $358) |  |  |
| 4 year post RT | $417 | $384 | -$33 | 0.38 |
|  | ($396 to $437) | ($316 to $451) |  |  |
|  |  |  |  |  |
| Urinary-Related Costs | |  |  |  |
| 1 year prior to RT | $1,074 | $1,114 | +$40 | 0.54 |
|  | ($1035 to $1112) | ($988 to $1239) |  |  |
| 1 year post RT | $1,648 | $1,511 | -$138 | 0.116 |
|  | ($1604 to $1693) | ($1352 to $1670) |  |  |
| 2 year post RT | $2,587 | $2,416 | -$171 | 0.225 |
|  | ($2525 to $2649) | ($2157 to $2675) |  |  |
| 3 year post RT | $3,541 | $3,094 | -$447 | 0.01 |
|  | ($3464 to $3618) | ($2787 to $3401) |  |  |
| 4 year post RT | $4,777 | $4,129 | -$648 | 0.002 |
|  | ($4676 to $4877) | ($3765 to $4493) |  |  |

RT, radiotherapy

The 100% Medicare dataset includes all Medicare patients, but excludes claims for office visits or from ambulatory surgery centers. Values are mean and (95% confidence interval) from Generalized Linear Models (GLMs) controlling for covariates (age, Charlson Comorbidity Index, modality, secondary cancer, baseline bowel/sexual/urinary dysfunction, data source, year of radiation therapy, and state fixed effects). Costs for the 1-year period prior to RT exclude the cost of the spacer. All costs “post” RT include all costs after the initiation of RT, excluding the cost of RT itself, and are cumulative.

**Supplemental Table 3**. Total Costs Over Time, by Insurer

|  | No Spacer (n=4,355) | Spacer  (n=224) | Difference | p-value |
| --- | --- | --- | --- | --- |
| Medicare 5% Data |  |  |  |  |
| 1 year prior to RT | $13,357 | $14,668 | +$1,311 | 0.085 |
|  | ($12926 to $13789) | ($13150 to $16185) |  |  |
| RT + RS | $20,753 | $23,014 | +$2,261 | <0.001 |
|  | ($20479 to $21027) | ($22020 to $24008) |  |  |
| 1 year post RT | $24,131 | $22,990 | -$1,141 | 0.42 |
|  | ($23543 to $24719) | ($20335 to $25645) |  |  |
| 2 years post RT | $33,520 | $31,497 | -$2,023 | 0.303 |
|  | ($32593 to $34447) | ($27857 to $35136) |  |  |
| 3 years post RT | $44,100 | $40,268 | -$3,832 | 0.13 |
|  | ($42701 to $45500) | ($35639 to $44897) |  |  |
| 4 years post RT | $54,976 | $50,692 | -$4,284 | 0.17 |
|  | ($53203 to $56748) | ($44894 to $56491) |  |  |
|  |  |  |  |  |
| MarketScan Data | No Spacer (n=1,204) | Spacer  (n=46) |  |  |
| 1 year prior to RT | $24,275 | $30,943 | +$6,668 | 0.046 |
|  | ($22725 to $25825) | ($23871 to $38015) |  |  |
| RT + Spacer | $56,103 | $68,517 | +$12,414 | 0.16 |
|  | ($53593 to $58614) | ($49829 to $87205) |  |  |
| 1 year post RT | $50,896 | $28,420 | -$22,477 | 0.001 |
|  | ($48221 to $53572) | ($18813 to $38027) |  |  |
| 2 years post RT | $59,885 | $32,830 | -$27,055 | <0.001 |
|  | ($56683 to $63087) | ($22557 to $43102) |  |  |
| 3 years post RT | $71,291 | $39,478 | -$31,813 | <0.001 |
|  | ($67070 to $75511) | ($27573 to $51383) |  |  |
| 4 years post RT | $83,205 | $48,210 | -$34,995 | <0.001 |
|  | ($78145 to $88265) | ($33713 to $62707) |  |  |

RT, radiotherapy; RS, rectal spacer

Values are mean and (95% confidence interval) from Generalized Linear Models (GLMs) controlling for covariates (age, Charlson Comorbidity Index, modality, secondary cancer, baseline bowel/sexual/urinary dysfunction, year of radiation therapy, and state fixed effects). Costs for the 1-year period prior to RT exclude the cost of the spacer. All costs “post” RT include all costs after the initiation of RT, excluding the cost of RT itself, and are cumulative.

**Supplemental Table 4**. Common Procedures (ICD-10) in 4 Years Post-RT by Spacer Status

|  |  | # per year per 1000 | | |
| --- | --- | --- | --- | --- |
| ICD10 | Description | No spacer | Spacer | Total |
| Bowel, Sexual, Urinary-related codes | |  |  |  |
| 0T9B70Z | Drainage of Bladder, Via Natural or Artificial Opening Endoscopic, Diagnostic. | 0.54 | 0.00 | 0.51 |
| 0VB08ZZ | Excision of Right Lower Leg Subcutaneous Tissue and Fascia, Open Approach. | 0.27 | 0.00 | 0.26 |
| 0TQB0ZZ | Repair Bladder, Open Approach. | 0.22 | 0.00 | 0.21 |
| 0T7D8ZZ | Dilation of Right Ureter, Via Natural or Artificial Opening Endoscopic Approach. | 0.18 | 0.00 | 0.17 |
| 0W3R8ZZ | Control Bleeding in Peritoneal Cavity, Via Natural or Artificial Opening Endoscopic Approach. | 0.13 | 0.93 | 0.17 |
| 0D1N0Z4 | Bypass Duodenum to Jejunum, Open Approach, Intraluminal Device. | 0.09 | 0.93 | 0.13 |
| 0DTP0ZZ | Resection of Sigmoid Colon, Open Approach. | 0.13 | 0.00 | 0.13 |
| 0V508ZZ | Destruction of Left Upper Leg Subcutaneous Tissue and Fascia, Endoscopic Approach. | 0.13 | 0.00 | 0.13 |
| 0VT08ZZ | Resection of Right Lower Leg Subcutaneous Tissue and Fascia, Open Approach. | 0.13 | 0.00 | 0.13 |
| 0DQB0ZZ | Repair Jejunum, Open Approach. | 0.09 | 0.00 | 0.09 |
| 0TPD0LZ | Removal of Intraluminal Device from Sigmoid Colon, Open Approach. | 0.09 | 0.00 | 0.09 |
| 0TUD07Z | Supplement Right Ureter with Autologous Tissue Substitute, Open Approach. | 0.09 | 0.00 | 0.09 |
| 0WQFXZ2 | Repair Left Hand Muscle, External Approach. | 0.09 | 0.00 | 0.09 |
| 0DBQ0ZZ | Repair Liver, Open Approach. | 0.04 | 0.00 | 0.04 |
| 0DQ88ZZ | Inspection of Small Intestine, Endoscopic Approach. | 0.04 | 0.00 | 0.04 |
| 0DTP4ZZ | Resection of Sigmoid Colon, Percutaneous Endoscopic Approach. | 0.04 | 0.00 | 0.04 |
| 0T9B30Z | Drainage of Bladder, Percutaneous Approach. | 0.04 | 0.00 | 0.04 |
| 0T9B80Z | Drainage of Bladder, Endoscopic Approach. | 0.04 | 0.00 | 0.04 |
| 0THD0LZ | Removal of Intraluminal Device from Right Ureter, Open Approach. | 0.04 | 0.00 | 0.04 |
| 0VPS0JZ | Removal of Synthetic Substitute from Left Lower Leg Subcutaneous Tissue and Fascia, Open Approach. | 0.04 | 0.00 | 0.04 |
| Codes unrelated to bowel, sexual, urinary dysfunction | |  |  |  |
| 0SRD0J9 | Replacement of Right Hip Joint with Synthetic Substitute, Open Approach. | 2.20 | 5.56 | 2.36 |
| 0SRC0J9 | Replacement of Left Hip Joint with Synthetic Substitute, Open Approach. | 2.20 | 3.70 | 2.27 |
| 0W3P8ZZ | Control Bleeding in Pelvic Cavity, Via Natural or Artificial Opening Endoscopic. | 1.75 | 2.78 | 1.80 |
| 4A023N7 | Measurement of Cardiac Output, Continuous, Less than 24 Hours. | 1.44 | 3.70 | 1.54 |
| 02HV33Z | Insertion of Pacemaker Lead into Right Ventricle, Percutaneous Approach. | 1.44 | 0.93 | 1.42 |
| 027034Z | Dilation of Coronary Artery, One Artery, with Drug-eluting Intraluminal Device, Percutaneous Approach. | 1.35 | 0.93 | 1.33 |
| 30233N1 | Transfusion of Nonautologous Red Blood Cells into Peripheral Vein, Percutaneous Approach. | 1.21 | 0.00 | 1.16 |
| 0DJ08ZZ | Inspection of Small Intestine, Open Approach. | 1.12 | 0.93 | 1.12 |
| 0FT44ZZ | Resection of Gallbladder, Percutaneous Endoscopic Approach. | 1.03 | 0.93 | 1.03 |
| 5A09357 | Respiratory Ventilation, Less than 24 Consecutive Hours, Continuous Positive Airway Pressure. | 0.90 | 1.85 | 0.94 |
| 0RRK00Z | Replacement of Right Knee Joint with Synthetic Substitute, Open Approach. | 0.90 | 0.00 | 0.86 |
| 02100Z9 | Bypass from Coronary Artery, One Artery to Abdominal Artery with Autologous Venous Tissue, Open Approach. | 0.67 | 2.78 | 0.77 |
| 01NB0ZZ | Release of Cranial Nerve, Open Approach. | 0.72 | 0.93 | 0.73 |
| 0JH606Z | Insertion of Spinal Cord Stimulator into Spinal Canal, Open Approach. | 0.67 | 0.93 | 0.69 |
| 5A1D70Z | Extracorporeal Photopheresis, Single. | 0.67 | 0.93 | 0.69 |
| 02RF38Z | Replacement of Pulmonary Valve with Zooplastic Tissue, Percutaneous Approach. | 0.63 | 0.00 | 0.60 |
| XW033E5 | Introduction of Remdesivir Anti-infective into Peripheral Vein, Percutaneous Approach, New Technology Group 5. | 0.58 | 0.93 | 0.60 |
| 0DBE8ZX | Excision of Liver, Via Natural or Artificial Opening Endoscopic Approach, Diagnostic. | 0.54 | 0.93 | 0.56 |
| 5A2204Z | Extracorporeal Hyperthermia, Whole Body. | 0.58 | 0.00 | 0.56 |
| 0DJD8ZZ | Inspection of Appendix, Via Natural or Artificial Opening Endoscopic Approach. | 0.54 | 0.00 | 0.51 |

**Supplemental Table 4b**. Total Number of Procedures (CPT) in 4 Years Post-RT by Spacer Status (per 1000 person-years)

|  | # per year per 1000 | |
| --- | --- | --- |
|  | No spacer | Spacer |
| Bowel-related procedures | 14.9 | 10.2 |
| Sexual-related procedures | 6.8 | 7.4 |
| Urinary-related procedures | 27.5 | 17.6 |

**Supplemental Table 5**. Number of Clinic Visits per Year by Spacer Status

|  | No Spacer (n=5,559) | Spacer (n=270) | Difference | p value |
| --- | --- | --- | --- | --- |
| All Visits |  |  |  |  |
| 1 year prior to RT | 32.97 | 32.98 | 0.01 | 0.99 |
| Annual average over 4 years post-RT | 29.6 | 28.8 | -0.87 | 0.018 |
| Bowel-related Visits |  |  |  |  |
| 1 year prior to RT | 0.10 | 0.14 | 0.04 | 0.092 |
| Annual average over 4 years post-RT | 0.33 | 0.28 | -0.05 | 0.22 |
| Sexual-related Visits |  |  |  |  |
| 1 year prior to RT | 0.31 | 0.40 | -0.09 | 0.035 |
| Annual average over 4 years post-RT | 0.21 | 0.23 | 0.03 | 0.38 |
| Urinary-related Visits |  |  |  |  |
| 1 year prior to RT | 1.59 | 1.67 | 0.09 | 0.32 |
| Annual average over 4 years post-RT | 1.42 | 1.25 | -0.17 | 0.036 |
| All Visits Unrelated to Bowel/Sexual/Urinary | |  |  |  |
| 1 year prior to RT | 31.0 | 30.7 | -0.23 | 0.55 |
| Annual average over 4 years post-RT | 27.7 | 27.0 | -0.72 | 0.042 |

RT, radiotherapy

Number of visits are based on Poisson regression estimation, controlling for covariates (age, Charlson Comorbidity Index, RT modality, secondary cancer, baseline bowel, sexual, urinary dysfunction, data source, year of RT, and state fixed effects). "Visits 1 year prior to RT" excludes visits for spacer insertion. Visits post RT is visits post the initiation of RT, excluding any visits related to RT, and are cumulative.

.

# Supplementary Figures

Panel A: Medicare 5% + MarketScan

Panel B: Medicare 100%

**Supplementary Figure 1.** Association between total cost of care and spacer use by type of RT modality. Panel A = Medicare 5% + MarketScan, Panel B = Medicare 100%. Positive coefficients indicate that the spacer use is associated with higher costs, and negative coefficients indicate that the spacer use is associated with lower costs. Coefficients are based on Generalized Linear Models (GLMs) controlling for covariates (age, Charlson Comorbidity Index, secondary cancer, baseline bowel/sexual/urinary disfunction, data source, year of radiation therapy, and state fixed effects). Costs for the 1-year period prior to RT exclude the cost of the spacer. Costs 4yr post RT include all costs after the initiation of RT, excluding the cost of RT itself, and are cumulative.
